# An Ultra‐Low Self‐Discharge Aqueous|Organic Membraneless Battery with Minimized Br_2_ Cross‐Over

**DOI:** 10.1002/advs.202307780

**Published:** 2024-01-03

**Authors:** Han Yang, Shiyu Lin, Yunpeng Qu, Guotao Wang, Shuangfei Xiang, Fuzhu Liu, Chao Wang, Hao Tang, Di Wang, Zhoulu Wang, Xiang Liu, Yi Zhang, Yutong Wu

**Affiliations:** ^1^ School of Energy Sciences and Engineering Nanjing Tech University Nanjing Jiangsu 211816 China; ^2^ College of Physics Guizhou University Guiyang 550025 China; ^3^ School of Materials Science and Engineering and Institute of Smart Fiber Materials Zhejiang Sci‐Tech University Hangzhou 310018 China; ^4^ State Key Laboratory for Mechanical Behavior of Materials Xi'an Jiaotong University Xi'an Shaanxi 710049 China; ^5^ School of Chemistry and Chemical Engineering Yangzhou University Yangzhou Jiangsu 225002 China

**Keywords:** battery self‐discharge, biphasic battery, membraneless battery, zinc bromine battery

## Abstract

Batteries dissolving active materials in liquids possess safety and size advantages compared to solid‐based batteries, yet the intrinsic liquid properties lead to material cross‐over induced self‐discharge both during cycling and idle when the electrolytes are in contact, thus highly efficient and cost‐effective solutions to minimize cross‐over are in high demand. An ultra‐low self‐discharge aqueous|organic membraneless battery using dichloromethane (CH_2_Cl_2_) and tetrabutylammonium bromide (TBABr) added to a zinc bromide (ZnBr_2_) solution as the electrolyte is demonstrated. The polybromide is confined in the organic phase, and bromine (Br_2_) diffusion‐induced self‐discharge is minimized. At 90% state of charge (SOC), the membraneless ZnBr_2_|TBABr (Z|T) battery shows an open circuit voltage (OCV) drop of only 42 mV after 120 days, 152 times longer than the ZnBr_2_ battery, and superior to 102 previous reports from all types of liquid active material batteries. The 120‐day capacity retention of 95.5% is higher than commercial zinc‐nickel (Zn–Ni) batteries and vanadium redox flow batteries (VRFB, electrolytes stored separately) and close to lithium‐ion (Li‐ion) batteries. Z|T achieves >500 cycles (2670 h, 0.5 m electrolyte, 250 folds of membraneless ZnBr_2_ battery) with ≈100% Coulombic efficiency (CE). The simple and cost‐effective design of Z|T provides a conceptual inspiration to regulate material cross‐over in liquid‐based batteries to realize extended operation.

## Graphical abstract

A low‐cost aqueous|organic battery based on the Zn–Br redox is designed with the assistance of tetrabutylammonium bromide to minimize Br_2_ shuttling‐induced self‐discharge. The 120‐day capacity retention of 95.5% without the presence of a physical membrane provides a conceptual inspiration to regulate material cross‐over in liquid‐based batteries and realize seasonal storage.

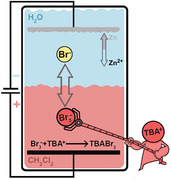

## 1 Introduction

The shift in global energy consumption has driven dramatic growth in renewable energy usage in the past decade, and intermittent energy sources, such as wind and solar, are targeted to provide more than 50% of the world's electricity supply by 2050.^[^ ^1^ ^]^ Weather, seasonal, and geographical factors all contribute to their fluctuating characteristics, creating inconsistencies between the energy provided and demand for stable supply/fast response over an extended timespan.^[^ ^2^, ^3^ ^]^ Investigating the next‐generation grid‐scale energy storage systems is thus necessary to balance the mismatch and unleash renewable energies' potential to a greater extent.^[^ ^4^, ^5^ ^]^

Batteries dissolving active materials in liquids (BLAM) possess safety and design flexibility advantages,^[^ ^6^, ^7^, ^8^ ^]^ thus playing an important role in serving as grid‐scale energy storage systems between traditional systems (e.g., pumped hydro) with larger sizes^[^ ^9^ ^]^ but geographically limited and smaller‐scale systems (e.g., Li‐ion batteries) with high energy density but safety concerns.^[^ ^1^, ^10^ ^]^ Different from solid active material‐based batteries (BSAM), despite the mode of the BLAM (static/flow) or the design (with/without membrane), the material cross‐over due to liquid nature remains a serious issue inducing self‐discharge both during battery cycling and idle when the electrolytes are in contact.

During cycling, for BLAM using non‐porous ion‐exchange membranes, such as all‐vanadium or organic redox, where there is no direct liquid‐liquid contact, ion cross‐over limits the electrochemical performance (especially the CE, and advanced active materials/membranes are being developed.^[^ ^11^ ^]^ In hybrid BLAM, such as Zn‐based systems, the combination of ion‐exchange membrane plus high‐concentration electrolyte additives can effectively reduce the cross‐over^[^ ^12^ ^]^ yet sacrifices with a drastic increase in the capital cost (≈50%).^[^ ^13^ ^]^ At the same time, the industry prefers cost‐effective porous membranes/membraneless designs^[^ ^14^ ^]^ in which cross‐over is inevitable.

During idle, it is often believed or promoted that BLAM can store electrolytes in separate reservoirs with “no self‐discharge.” However, the cross‐over during electrolyte cycling and the levelized cost from electrolyte cycling (≈4.7%)^[^ ^15^, ^16^ ^]^ is difficult to neglect, especially at the grid scale. Interestingly, a self‐discharge of 0.05% per day was reported from a commercial 250 kW/1000 kWh VRFB with electrolytes stored separately.^[^ ^17^ ^]^ The strategy to pump and store electrolytes also leads to an increased footprint and limits the system response time.^[^ ^18^ ^]^

BLAMs are thus typically applied to daily operations with hourly‐based energy storage/distribution durations,^[^ ^19^ ^]^ hindering their wide applications over an extended time and against other technologies. We promote here the concept of an aqueous|organic membraneless battery based on the Zn–Br redox,^[^ ^20^ ^]^ namely the Z|T battery, as a demonstration of regulating cross‐over through two immiscible phases through an incredibly long timespan with low cost. The Br_2_ cross‐over induced self‐discharge was minimized through an extremely simple strategy—dissolving TBABr complexed solid polybromides into CH_2_Cl_2_ and confining Br_2_ in the organic phase.

## 2 Results and Discussion

**Figure 1:**
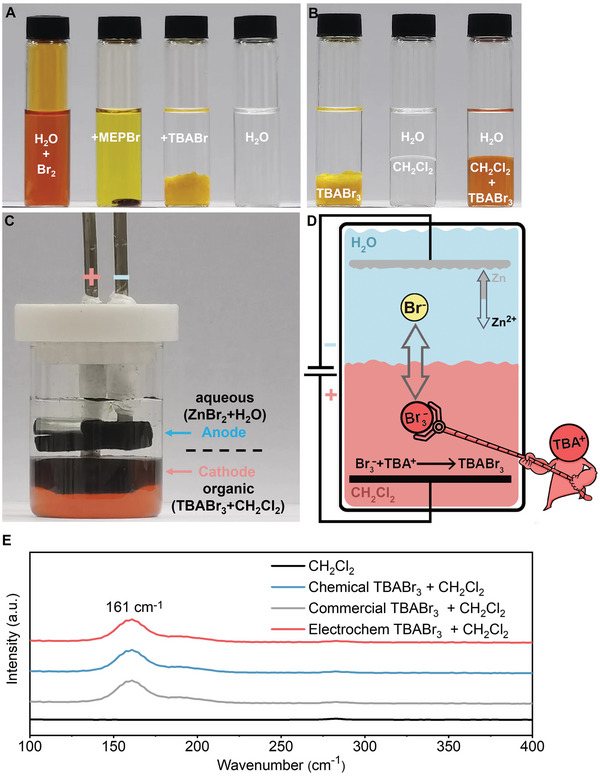
Electrolyte characteristics and Z|T battery assembly. A) Photos of 0.2 m bromine water, MEPBr‐added 0.2 m bromine water, TBABr‐added 0.2 m bromine water, and deionized water (DI water) from left to right. B) Photos of TBABr‐added 0.2 m Br_2_, 1:1 volume ratio of DI water to CH_2_Cl_2_, 1:1 volume ratio of DI water to TBABr‐added CH_2_Cl_2_ from left to right. C) Photo of a Z|T battery at 90% SOC. D) Z|T battery schematic, TBABr_3_ forms during charging. E) Raman spectra of CH_2_Cl_2_, chemically generated TBABr_3_ (0.5 m TBABr + 0.45 m Br_2_) in CH_2_Cl_2_, commercial TBABr_3_ (0.45 m) in CH_2_Cl_2_ and electrochemically generated TBABr_3_ (0.5 m Z|T charged to 90% SOC).

TBABr complexed volatile Br_2_ into water‐insoluble products,^[^ ^21^, ^22^, ^23^ ^]^ different from the conventional complexing agent *N*‐methyl‐*N*‐ethyl‐pyrrolidinium bromide (MEPBr) used in ZnBr_2_ batteries, which formed phase‐separating oil‐like polybromides (**Figure** **1**A).^[^ ^24^ ^]^ While MEPBr lowered the Br_2_ dissolution in water, minor polybromide still existed in the electrolyte to cause Br_2_ cross‐over, evident from the yellow supernatant; the transparent supernatant for the hydrophobic TBABr did not show a sign of polybromide existence. The supernatants were collected, then added with additional TBABr, and tested with potassium iodide starch test paper (KI paper) to further confirm the statement. MEPBr‐added supernatant formed extra solid polybromides after TBABr addition and KI paper turned blue, while TBABr‐added supernatant neither formed polybromides nor did KI paper change color, supporting the claim (Figure S1, Supporting Information). Although TBABr exhibited an outstanding ability to prevent self‐discharge, it is unsuitable for typical BLAMs since the insoluble products formed detach from the electrode and lead to capacity fade.^[^ ^21^ ^]^

Water‐immiscible and higher‐density CH_2_Cl_2_ was used to support TBABr_3_, which kept the organic phase below the aqueous phase to avoid volatility‐induced safety issues. As a result, an aqueous|organic system was formed when CH_2_Cl_2_ was added to water, and the aqueous phase stayed transparent after adding TBABr_3_ (Figure 1B). Biphasic membraneless ZnBr_2_|TBABr batteries were designed so that the TBABr concentration and organic phase volume varied based on the ZnBr_2_ concentration in the aqueous phase (TBABr:Br_2_ ≥ 1:1). Electrodes were immersed into the two phases separately. Figure 1C shows a 0.5 m Z|T battery (0.5 m refers to the aqueous phase concentration) at a 90% SOC. Bromide (Br^−^) in the aqueous phase was oxidized to Br_2_ on the cathode and complexed by TBABr to form TBABr_3_ in the organic phase during charging, while metallic zinc (Zn) was formed on the anode (Figure 1D). To verify that no side products/reactions occurred, Raman spectroscopy was conducted for 1) commercial TBABr_3_ in CH_2_Cl_2_, 2) chemically formed TBABr_3_ by adding Br_2_ to TBABr in CH_2_Cl_2_, and 3) electrochemically generated TBABr_3_ through 0.5 m Z|T charged to 90% SOC. The signature peak representing the symmetric stretching mode of tribromide (Br_3_^−^) at 161 cm^−1[^ ^21^ ^]^ was shown in all three samples (Figure 1E), evidencing that TBABr_3_ was successfully confined in the organic phase through charging.

**Figure 2:**
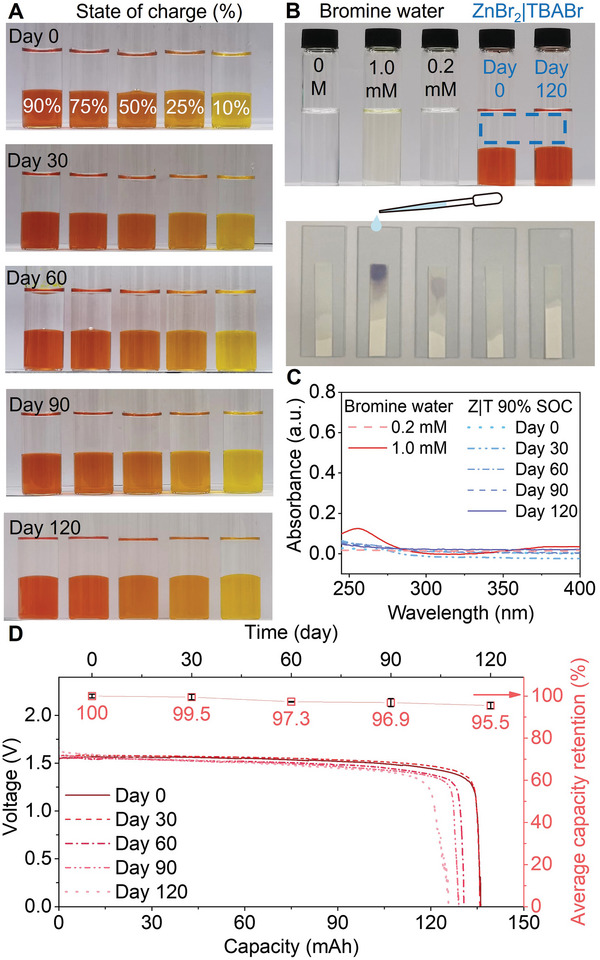
Z|T 120‐day stability and capacity retention. A) Photos of 0.5 m Z|T electrolytes at 10–90% SOCs from 0–120 days. B) KI paper test on DI water, Br_2_ water (1 and 0.2 mm), and the aqueous phase of 0.5 m Z|T (Days 0 and 120) at 90% SOC, KI paper turns blue if trivial Br_2_ exists, also see Movie S1, Supporting Information. C) UV–vis spectra of Br_2_ water (1 and 0.2 mm) and the aqueous phase of 0.5 m Z|T (Days 0, 30, 60, 90, and 120) at 90% SOC. D) Capacity retention of 0.5 m Z|T on Days 0, 30, 60, 90, and 120 (independent repeat samples, see Figure S3, Supporting Information), error bars represent mean ± standard deviation, sample size (*n*) = 3.

To confirm that the ultra‐low self‐discharge stands in a long time span for the Z|T systems, a series of characterizations were conducted on the aqueous electrolyte. First, no visible differences or Br_2_ cross‐over were observed from 10–90% SOC electrolytes (0.5 m) stored after 0, 30, 60, 90, and 120 days (**Figure** **2**A). A trace amount of Br_2_ (indistinguishable against pure water from the naked eye) was difficult to qualify, yet we found the KI paper surprisingly effective in verifying Br_2_'s existence in solutions. The aqueous phase electrolytes of 0.5 m Z**|**T at 90% SOC stored for 0 and 120 days were tested with KI paper and did not show a blue color, while 1 and 0.2 mm bromine water did (Figure 2B and Movie S1, Supporting Information). KI paper was unable to detect <0.005 mm Br_2_ in water with blue color change (0.001% of 0.5 m Z|T total capacity), a concentration not of significant concern for battery applications. Ultraviolet‐visible (UV–vis) absorption peak (Figure 2C) was observed near 270 nm, the polybromide ion peak^[^ ^25^ ^]^ for the 1 mm Br_2_ sample and not for the 0.5 m Z|T 0–120 days aqueous phase samples, further confirming the absence of Br_2_ in the aqueous phase. The pH value of the aqueous phase also remained relatively stable at different SOCs (Figure S2, Supporting Information), in comparison, the pH dropped to 4.05 when a trivial amount of Br_2_ (1 mm) was added to water.

The long‐term capacity loss was then tested, and 0.5 m Z|T at 90% SOCs were stored and discharged on days 0–120. Taking the capacity on Day 0 as 100%, Z|T maintained a capacity retention of 95.5% even after 120 days (Figure 2D). The capacity retention averaged three individual samples at each time point, and Figure S3, Supporting Information, presents the rest of the electrochemical experiments. The results imply that the organic phase strongly inhibited bromine cross‐over in long‐term storage even at high SOCs, the Z|T electrolyte was successfully stored for more than 4 months, even when the aqueous|organic electrolytes were in contact without any external assistance such as splitting the electrolytes in separate reservoirs.

**Figure 3:**
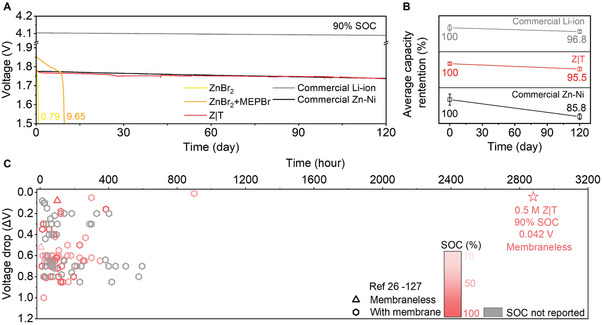
Z|T 120‐day OCV retention V.S. commercial batteries and 102 references. A) OCV measurements of 0.5 m ZnBr_2_, ZnBr_2_+MEPBr, and Z|T; commercial BSAM Li‐ion and Zn–Ni batteries (other samples are in Figures S5 and S6, Supporting Information). B) Capacity retention of 0.5 m Z|T, commercial Li‐ion, and Zn–Ni batteries at 90% SOC (independent repeat samples, Figure S7, Supporting Information), error bars represent mean ± standard deviation, *n* = 3 except for Day 120 Li‐ion and Day 0 Zn–Ni batteries (*n* = 2). C) OCV retention comparison of 0.5 m Z|T at 90% SOC to 102 previously reported BLAMs^[^ ^26^, ^27^, ^28^, ^29^, ^30^, ^31^, ^32^, ^33^, ^34^, ^35^, ^36^, ^37^, ^38^, ^39^, ^40^, ^41^, ^42^, ^43^, ^44^, ^45^, ^46^, ^47^, ^48^, ^49^, ^50^, ^51^, ^52^, ^53^, ^54^, ^55^, ^56^, ^57^, ^58^, ^59^, ^60^, ^61^, ^62^, ^63^, ^64^, ^65^, ^66^, ^67^, ^68^, ^69^, ^70^, ^71^, ^72^, ^73^, ^74^, ^75^, ^76^, ^77^, ^78^, ^79^, ^80^, ^81^, ^82^, ^83^, ^84^, ^85^, ^86^, ^87^, ^88^, ^89^, ^90^, ^91^, ^92^, ^93^, ^94^, ^95^, ^96^, ^97^, ^98^, ^99^, ^100^, ^101^, ^102^, ^103^, ^104^, ^105^, ^106^, ^107^, ^108^, ^109^, ^110^, ^111^, ^112^, ^113^, ^114^, ^115^, ^116^, ^117^, ^118^, ^119^, ^120^, ^121^, ^122^, ^123^, ^124^, ^125^, ^126^, ^127^ ^]^ (with/without membrane, static/flow) at various SOCs corresponding to Table S1, Supporting Information.

OCV test is a common and effective method for continuous battery self‐discharge monitoring on both lab and industrial scales.^[^ ^25^ ^]^ It is especially suitable for BLAMs since they have rather flat voltage profiles, thus was conducted to assess Z|T battery self‐discharge fairly. The OCV of 0.5 m ZnBr_2_ batteries, namely 1) the ZnBr_2_ battery (no complexing agent, single phase), 2) the ZnBr_2_+MEPBr battery (with a complexing agent, single phase, the common approach), and 3) Z**|**T (biphasic) were compared without membrane at 90% SOC. First, the OCV of the ZnBr_2_ battery decreased drastically after only 0.79 days due to rapid Br_2_ cross‐over without restrictions (**Figures** **3**A and S4A,B, Supporting Information), while ZnBr_2_+MEPBr maintained 9.65 days before a rapid voltage decay (Figure 3A and Figure S4C,D, Supporting Information), indicating that Br_2_ cross‐over was severe even with MEPBr as the complexing agent when a physical membrane was not applied. In contrast, the OCV of Z|T only decayed for 42 mV even after 120 days (Figure 3A and Figure S5, Supporting Information), 152 and 12.4 times longer than ZnBr_2_ and ZnBr_2_+MEPBr batteries, not to mention the negligible voltage drop, demonstrating an unparalleled self‐discharge advantage against typical ZnBr_2_ batteries.

The OCV profiles of widely used commercial BSAM products, namely Li‐ion and Zn–Ni batteries, were monitored and compared (Figure 3A and Figure S6, Supporting Information). 0.5 m Z|T at 90% SOC showed a similar voltage decay to commercial Zn–Ni batteries (35 mV) and was slightly higher than commercial Li‐ion batteries (14 mV). Moreover, the 95.5% 120‐day capacity retention Z|T was higher than commercial Zn–Ni batteries (85.8%) and comparable with commercial Li‐ion batteries (96.8%, Figure 3B and Figure S7, Supporting Information). The 0.0375% per day capacity retention of Z|T was even lower than that of commercial VRFB (0.05% per day) with electrolytes stored in separate reservoirs.^[^ ^17^ ^]^ Note that the OCV drop value across Z|T and commercial BSAMs is for reference since the voltage profiles could be different, and the repeatable capacity retention data based on multiple trials represents the self‐discharge status.

Z|T OCV retention was then compared to 102 previously reported BLAM OCV retention data, and most batteries exhibited >100 mV OCV drop for <17 days at ≤75% SOCs (Figure 3C and Table S1, Supporting Information). Note that for most of the references, the research focus was advanced membrane material development, and commercial Nafion membranes were used in their control samples. The long‐term self‐discharge performance was merely mentioned for biphasic membraneless batteries, and 80 h with 62.4 mV from a fully charged state for Zn|2,2,6,6‐tetramethyl‐1‐piperidinyloxy (methyl cyanide)^[^ ^26^ ^]^ was the longest reported, as we know. The results suggested that the Z|T could stably store at high SOCs for months without external assistance or electrolyte separation.

**Figure 4:**
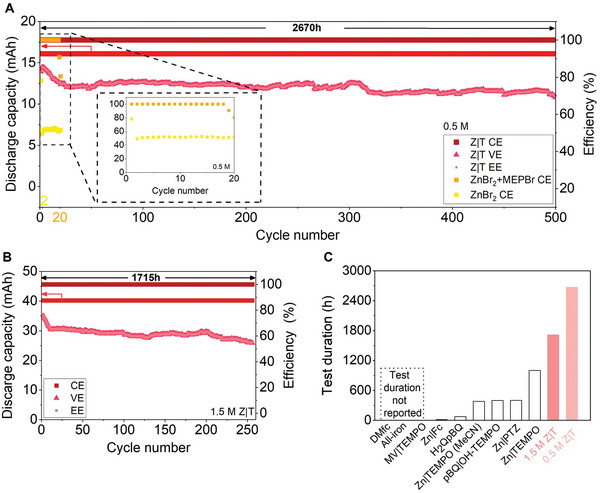
Z|T long‐term cycling performance and comparison to other membraneless batteries. A) 0.5 m Z|T discharge capacity, efficiencies, and test duration, other samples are in Figure S8, Supporting Information; the inset shows the CE of ZnBr_2_ and ZnBr_2_+MEPBr batteries, other samples are in Figure S10, Supporting Information. B) 1.5 m Z|T discharge capacity, efficiencies, and test duration, other samples are in Figure S12, Supporting Information. C) Comparison of total test time between current biphasic membraneless batteries^[^ ^26^, ^127^, ^129^, ^130^, ^131^, ^132^, ^133^, ^134^, ^135^ ^]^ corresponding to Table S2, Supporting Information.

During BLAM cycling and when the electrolytes are in direct contact, cross‐over induced self‐discharge can cause severe efficiency decay and greatly influence performance. The 0.5 m ZnBr_2_ batteries mentioned above were cycled at 2 mA cm^−2^, and the batteries were pre‐charged to 50% SOC and cycled with 10% total volumetric capacity (2.68 Ah L^−1^) for each cycle without membranes. 0.5 m Z|T achieved 100% capacity retention over 500 cycles, with nearly 100% CE and 73.9% average energy efficiency (EE) (**Figures** **4**A and S8, Supporting Information). A stable voltage profile was maintained after 1000 h of battery operation with minimum polarization (Figure S9, Supporting Information). In contrast, the CE of the ZnBr_2_ battery quickly decreased to 49.5% in only two cycles, and the CE of the ZnBr_2_+MEPBr battery dropped to 80.4% in only 20 cycles (Figure 4A and Figure S10, Supporting Information). Figure S11A, Supporting Information, demonstrates an obvious capacity decay of the ZnBr_2_ to less than half of the charged capacity in the third cycle for ≈12 h, while the ZnBr_2_+MEPBr stopped quickly after the 20th cycle at a little more than 100 h suggested by the voltage profile (Figure S11B, Supporting Information). The cycle life of Z|T was 250 folds of the ZnBr_2_ battery, and the total cycling duration of Z|T was approaching 4 months (2670 h) thanks to minimized Br_2_ cross‐over since the metallic Zn formed during cycling could have been easily corroded by Br_2_ diffusion in only hours. The advantage of Zn–Br redox against other BLAMs is the higher theoretical energy density due to high ZnBr_2_ solubility in water,^[^ ^128^ ^]^ thus 1.5 m Z|T (8.04 Ah L^−1^) with three folds energy densities were also tested, and maintained ≈100% CE and 64.04% average EE for >256 cycles (1715 h, Figure 4B and Figure S12, Supporting Information) at doubled current density. Although being more polarized compared to the 0.5 Z|T cycling due to increased electrolyte concentration/current density, 1.5 Z|T also had a stable voltage profile even after 1275 h of battery cycling (Figure S13, Supporting Information). The results indicate that the Z|T concept with minimized self‐discharge can be well adapted to various Zn–Br redox concentrations without a physical membrane. Besides the longest cycling duration achieved (0.5 m Z|T), both the volumetric capacity and cycle number of 0.5 and 1.5 m Z|T were on the top tier among all biphasic membraneless batteries (Figure 4C and Table S2, Supporting Information).

Note that we intentionally avoided pretreatments to any materials used and did not add electrolyte additives so that the minimized Br_2_ cross‐over was achieved solely through the CH_2_Cl_2_+TBABr idea. The descending EE at increased ZnBr_2_ concentration was due to metallic Zn formation in the Z|T aqueous phase with increased energy and current densities per cycle, and Zn dendrite formation and low utilization during cycling were the major issues while no Br_2_ cross‐over was observed. We also admit that the lower current density compared to mature BLAM systems like flow or single‐phase batteries is a common issue for biphasic batteries due to the low ion conductivity in the organic phase and needs improvements.^[^ ^132^ ^]^ However, considering the static and membraneless design within a small 20 mL vial as the battery case, Z|T's cycling performance was incredible since the >2500 h cycling is a Br_2_ cross‐over controlled process, evidenced by the rapid fade in the OCV and CE of the ZnBr_2_ and ZnBr_2_+MEPBr batteries in <300 h.

Preventing Br_2_ cross‐over through phase separation and confining Br_2_ away from Zn was the core concept to minimize self‐discharge. To verify this further, we created an extreme situation where metallic Zn formed, accumulated, and filled the aqueous phase and was in contact with TBABr_3_ on the aqueous|organic interface with high ZnBr_2_ utilization (0.5 m Z|T, 90% SOC). The average CE of 95.9% (Figure S14, Supporting Information) indicated that Br_2_ corroded Zn and led to minor Zn consumption and dendrite formation.^[^ ^136^, ^137^, ^138^ ^]^ Note that although only ten cycles were realized, the long cycling duration of 628 h again confirmed that Z|T still survived long in the Br_2_ cross‐over controlled process. Another advantage of Z|T compared to other biphasic membraneless batteries is that it is unnecessary to undergo additional functional group modification to suppress charged species cross‐over to the aqueous phase.^[^ ^139^, ^140^, ^141^ ^]^

The Z|T battery possesses unparalleled advantages in the capital cost due to the abundant and low‐cost ZnBr_2_,^[^ ^16^ ^]^ along with the common industrial solvent CH_2_Cl_2_^[^ ^130^ ^]^ and the classic organic synthesis catalyst TBABr.^[^ ^142^, ^143^, ^144^ ^]^ The capital cost of Z|T calculated with the conventional method is only $18.9/kWh. We have also provided an unconventional, yet convincing electrolyte cost analysis based on the prices from 3 major lab‐scale chemical reagent suppliers since some active materials from other biphasic membraneless batteries are difficult to obtain on the industrial scale. Setting the price of 0.5 m Z|T in $/kWh to be 1, despite the regional and currency differences, the three comparisons follow the same trend that the prices of other biphasic membraneless batteries are 5.6–1071.9 times higher than that of 0.5 m Z|T, with Zn|PTZ as the lowest and H_2_Q|pBQ being the highest (Figure S15 and Tables S3–S5, Supporting Information). Details of the cost analysis can be found in the Supporting Information (Table S6, Supporting Information). Note that the vanadium electrolyte+membrane cost was 1.5–4.5 folds of 0.5 m Z|T.

Despite the astounding Br_2_ cross‐over related performance in the current conceptual demonstration, various aspects can be optimized to help improve Z|T energy/current densities and overall performance. For the aqueous phase, metallic Zn growth‐related issues, such as dendrites, can be regulated with natural pH Zn dendrite suppression strategies^[^ ^145^ ^]^ or switched to a metal‐free design.^[^ ^146^ ^]^ For the organic phase, while CH_2_Cl_2_ is one of the typical solvents used for biphasic membraneless batteries, low ion conductivity can be improved through additives,^[^ ^130^ ^]^ and the storage and safety concerns can be addressed through device modification based on the commercial CH_2_Cl_2_ container.^[^ ^147^, ^148^ ^]^ The theoretical energy density could be increased through organic solvent selection plus functional group tuning on the complexing agent.^[^ ^149^ ^]^ On the other hand, advanced electrode materials such as phase‐affinity‐tuned carbon felt^[^ ^150^ ^]^ and device engineering^[^ ^151^ ^]^ would promote electrochemical properties.

## 3 Conclusion

The Z|T prototype battery paves the way for BLAMs to realize an ultra‐low self‐discharge both during battery cycling and idle when electrolytes are in direct contact through its unique biphasic membraneless design that minimizes Br_2_ cross‐over. The exceptionally low self‐discharge provides the opportunity for BLAMs to store electrolytes at a high SOC for months and explore the option for stepwise utilization to expand their daily operational modes to a longer time span (E.g., charge the battery to 90% SOC in August and discharge in December). The simplicity of Z|T battery design and assembly, as already demonstrated above, makes it easy to conduct repeated experiments at the lab scale for fundamental understanding and performance optimization. At the same time, the cost‐effectiveness promotes Z|T's high scalability to industrial applications with a reduced footprint.

## Conflict of Interest

Y.W., X.L., Y.Z., H.Y., and S.L. are the inventors of patent CN2022116091217 (in application). The remaining authors declare no conflict of interest.

## Author Contributions

H.Y., S.L., and Y.Q. contributed equally to this work. H.Y. and S.L. assembled Z|T batteries and conducted battery tests. Y.Q. and G.W. performed initial biphasic electrolyte experiments. H.Y., C.W., and H.T. tested commercial batteries. S.L., Y.Q., and D.W. characterized the electrolytes. P.X., F.L., and Z.W. summarized the OCV data and calculated the cost comparisons. Y.W. designed Z|T batteries, and Y.Z., X.L., and Y.W. directed all experiments and characterizations. H.Y., S.L., Y.Q., Y.Z., X.L., and Y.W. contributed to manuscript drafting.

## Supporting information

Supporting Information

Supplemental Movie 1

## Data Availability

The data that support the findings of this study are available from the corresponding author upon reasonable request.
