# Supplementary material for: An Ultra‐Low Self‐Discharge Aqueous|Organic Membraneless Battery with Minimized Br2 Cross‐Over
Source: Adv Sci (Weinh). 2024 Jan 3;11(7):2307780. doi: 10.1002/advs.202307780 (PMC10870083; doi:10.1002/advs.202307780)
Supplement: Supplementary file 1 — Supporting Information [file ADVS-11-2307780-s002.pdf]

ADVANCED SCIENCE Open Access

Supporting Information

for *Adv. Sci.*, DOI 10.1002/advs.202307780

An Ultra-Low Self-Discharge Aqueous|Organic Membraneless Battery with Minimized Br_2 Cross-Over

Han Yang, Shiyu Lin, Yunpeng Qu, Guotao Wang, Shuangfei Xiang, Fuzhu Liu, Chao Wang, Hao Tang, Di Wang, Zhoulu Wang, Xiang Liu, Yi Zhang* and Yutong Wu**

Supplementary Information for

An Ultra-Low Self-Discharge Aqueous|Organic Membraneless Battery with Minimized Br_2 Cross-over

Han Yang[1,+], Shiyu Lin[1,+], Yunpeng Qu[2,+], Guotao Wang[1], Shuangfei Xiang[3], Fuzhu Liu[4], Chao Wang[5], Hao Tang[1], Di Wang[1], Zhoulu Wang[1], Xiang Liu[1,*], Yi Zhang[1,*] and Yutong Wu[1,*]

[1]School of Energy Sciences and Engineering, Nanjing Tech University, Nanjing 211816, Jiangsu, China
[2]College of Physics, Guizhou University, Guiyang, 550025, China.
[3]School of Materials Science and Engineering and Institute of Smart Fiber Materials, Zhejiang Sci-Tech University, Hangzhou 310018, China.
[4]State Key Laboratory for Mechanical Behavior of Materials, Xi'an Jiaotong University, Xi'an 710049, Shaanxi, China
[5]School of Chemistry and Chemical Engineering, Yangzhou University, Yangzhou 225002, Jiangsu, China

[+]These authors contributed equally to this work.

*Correspondence to:
iamxliu@njtech.edu.cn (X. Liu);
zhangy@njtech.edu.cn (Y. Zhang);
yw19@njtech.edu.cn (Y. Wu)

This file includes:

Materials and Methods (Pages S2-S3)
Figures S1 to S15 (Pages S4-S13)
Tables S1 to S6 (Pages S14-S21)
Movie S1 (Pages S21)
References (Pages S22)

Materials and methods

Chemicals and materials

All chemicals were used as received without any pretreatments. Zinc bromide ($ZnBr_2$, 99%, Macklin), N-methyl-N-ethyl-pyrrolidinium bromide (MEPBr, 99%, Macklin), tetrabutylammonium bromide (TBABr, 99%, Aladdin), tetrabutylammonium tribromide ($TBABr_3$, 98%, Aladdin), graphite felt (GF, G600A, SCI Materials Hub), potassium iodide starch test paper (Shanghai SSS Reagent Co., Ltd.), commercial Li-ion battery (3400 mAh, NCR18650B, Panasonic Energy Co., Ltd.), commercial Zn-Ni battery (1250 mAh, Delipow Battery Technology Co., Ltd.).

Battery assembly

"|" represents biphasic membraneless batteries and "+" indicates single phase batteries with additives.

For all $ZnBr_2$ based batteries, graphite felt (2 cm × 1.5 cm × 0.6 cm) was immersed into the organic phase to serve as the cathode, graphite felt (2 cm × 1 cm × 0.6 cm) enveloped with a zinc foil (2 cm × 1 cm × 0.05 cm) was used as the anode in the aqueous phase, with titanium wires (diameter: 3 mm) as the current collector. The Titanium wires were covered with PTFE tapes, so they were not exposed to electrolytes.

$ZnBr_2$|TBABr battery (0.5 M) was assembled with 6 mL of 0.5 M $ZnBr_2$ in DI water as the anolyte, and 6 mL of 0.5 M TBABr in CH_2Cl_2 was used as the catholyte in a 20 mL glass vial (diameter: 2.6 cm), the total capacity (100% SOC) was 160.8 mAh. Z|T (1.5 M) was assembled with 5 mL of 1.5 M $ZnBr_2$ in DI water as the anolyte and 9.375 mL of 0.8 M TBABr/CH_2Cl_2 was used as the catholyte in a 30 mL glass vial (diameter: 2.6 cm), the total capacity was 402 mAh. Organic and aqueous electrolytes were added to the glass vial successively. $ZnBr_2$ battery was assembled with 6 mL of 0.5 M $ZnBr_2$ in DI water as the electrolyte without any additives in a 20 mL vial. $ZnBr_2$+MEPBr battery was assembled with 6 mL of 0.5 M $ZnBr_2$ in DI water with 0.5 M MEPBr additive as the electrolyte in a 20 mL vial. The two electrodes were positioned in the same configuration as the Z|T batteries.

Characterizations

MEPBr (0.2 M, 0.466 g) and TBABr (0.2 M, 0.774 g) were added to 12 mL of 0.2 M bromine water (Figure 1A). An additional 0.774 g TBABr was added to 6 mL of MEPBr-added and TBABr-added supernatant from Figure 1 (Figure S1). Liquids were drawn and pipetted onto KI papers to qualify Br_2 existence through color change. Note that commercial $TBABr_3$ contains Br_2 from the KI paper test, since the synthesis protocol was not specified, we hypothesize that Br_2 vapor was used. Thus, the "no KI paper color change" only applied to electrochemically/chemically (Br_2 liquid mixing with commercial TBABr) generated $TBABr_3$ with TBABr : $Br_2 \geq 1:1$. Raman spectrum (DXR, Thermo Fisher) was recorded with a laser wavelength of 532 nm and a spectral range of 100-400 cm^{-1} (Figure 1E). 0.45 M Br_2 and 0.5 M TBABr were added into CH_2Cl_2 as the "Chemical $TBABr_3$ + CH_2Cl_2" sample; 0.45 M commercial $TBABr_3$ dissolved in CH_2Cl_2 served as the "Commercial $TBABr_3$ + CH_2Cl_2" sample; the "Electrochem $TBABr_3$ + CH_2Cl_2" sample was collected from the organic phase of 0.5 M Z|T at 90% SOC. Samples (Day 0, 30, 60, 90 and 120) of Z|T aqueous phase electrolytes were tested with UV-vis (Shimadzu, UV-3600i Plus) along with 0.2 and 1.0 mM bromine water without dilution (Figure 2C).

Electrochemical measurements

For the OCV retention tests, all batteries were charged to 90% SOC, and the voltage profiles were monitored and recorded from Day 0 to 120, with two or more samples for each type of battery. The $ZnBr_2$ based batteries were charged to 144.72 mAh with a cutoff voltage of 2.1 V at 2 mA/cm^2.

0.01 cm thick Zn foils instead of 0.05 cm were used to accelerate the bromine cross-over effect on Zn. The commercial Li-ion batteries were charged to 3060 mAh, and the Zn-Ni batteries were charged to 1125 mAh. For the capacity retention tests, the 0.5 M 90% SOC Z|T batteries were discharged at 1 mA/cm^2 (0.0187C) on Days 0, 30, 60, 90 and 120 with a 0 V cutoff voltage or 144.72 mAh capacity. Commercial Li-ion batteries at 90% SOC were discharged at a current of 50 mA (0.0147C) on Days 0 and 120 with a cutoff voltage of 2.75 V or a capacity of 3060 mAh. Commercial Zn-Ni batteries at 90% SOC were fully discharged at a current of 20 mA on Days (0.0160C) and 120 with a cutoff voltage of 1.4 V or a capacity of 1125 mA. 3 repeated tests were conducted for each type of battery.

For the cycling tests, the 0.5 M Z|T, $ZnBr_2$ and $ZnBr_2$+MEPBr batteries were pre-charged to 50% SOC and cycled at a constant current density (2 mA/cm^2) with a capacity corresponding to 10% total capacity for each cycle. 1.5 M Z|T was pre-charged to 50% SOC and cycled at 4 mA/cm^2 with 10% total capacity for each cycle. For the deep cycling tests, the 0.5 M Z|T battery was cycled at 1.5 mA/cm^2, with 90% total capacity for each cycle. The cutoff voltages were 2.1 V and 0 V, and 3 repeated tests were conducted for each type of battery.

The battery capacity was calculated using Equation S1.

$$C = n \cdot e^{-} \cdot F \qquad \text{S1}$$

n → amount of active species (mol)

e^- → electrons transferred per mole of storage compound

F → Faraday constant (96485 A·s mol^{-1})

For 0.5 M Z|T, since the active material $ZnBr_2$ was confined to the aqueous phase and the organic phase only served as the reservoir for the cathode side charged product, the aqueous phase was thus used for battery capacity calculation, the same as other biphasic batteries. C = (0.006 L · 0.5 mol L^{-1} · 2 · 96485 A·s mol^{-1})/3.6=160.8 mAh).

Cost calculation

The chemicals and reagents prices of other biphasic membraneless and vanadium batteries were referenced from three major suppliers, namely Aladdin (Supplier 1), Sigma-Aldrich (Supplier 2) and Alfa Aesar (Supplier 3). The cost was calculated based on Equation S2. Since the prices of lab scale reagents may vary significantly on the purity and quantity, the price of 100 g reagent was used, and the mass was rounded up to the nearest value (less expensive) if 100 g was not available. For reagent purity, the same purity was selected from the three suppliers for a fair comparison. If a specific purity was unavailable from a supplier, the price was rounded down to the price of the nearest purity (no less than 98%), while for Z|T the price was rounded up. The prices from the other two suppliers were used if individual reagents were unavailable for a specific supplier. A membrane cost of 27.4%[1] was accounted for vanadium flow batteries.

$$S = \frac{\sum(P \cdot m)}{C \cdot V} \qquad \text{S2}$$

S → Battery cost ($/kWh)

P → Price of each reagent ($/kg)

m → Mass of each reagent (kg)

C → Battery capacity (Ah)

V → Battery voltage (V)

Statistical analysis

Origin was used for statistical analysis and no data pre-processing was conducted, the original data for the statical analysis in Figures 2D and 3B are presented in Figures S3 and S7. The sample size (n) is 3 for most cases, except for Day 120 commercial Li-ion battery capacity and Day 0 (Figure S7B) commercial Zn-Ni capacity (Figure S7C), where n=2. The mean was calculated for Day 0 to 120 in Figure 2D, and 100% average capacity retention referred to the mean of Day 0 discharge capacity. The error bar was presented in the format of mean ± standard deviation. The analysis in Figure 3B was performed in the same fashion as in Figure 2D.

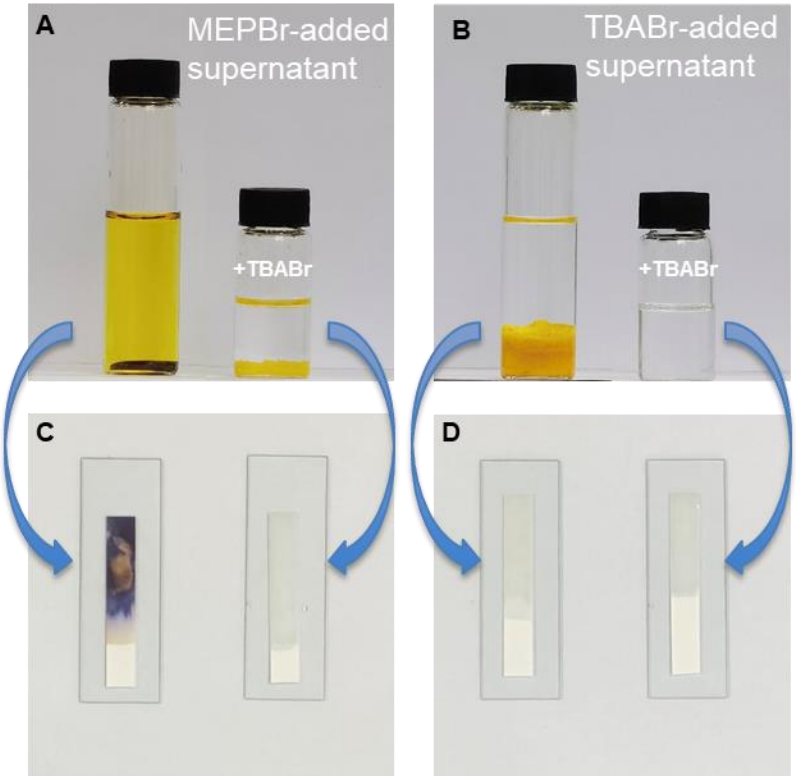

Figure S1. Adding additional TBABr and KI paper to qualify Br_2 existence in (A) MEPBr-added supernatant (B) TBABr-added supernatant. (C) KI paper results for MEPBr-added supernatant (D) KI paper results for TBABr-added supernatant.

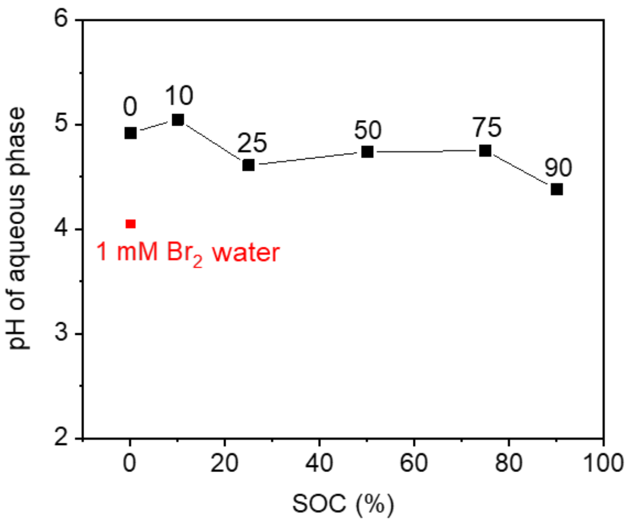

Figure S2. The pH value of the 0.5 Z|T aqueous phase at various SOC ranging from 0% to 90% and 1 mM Br_2 water.

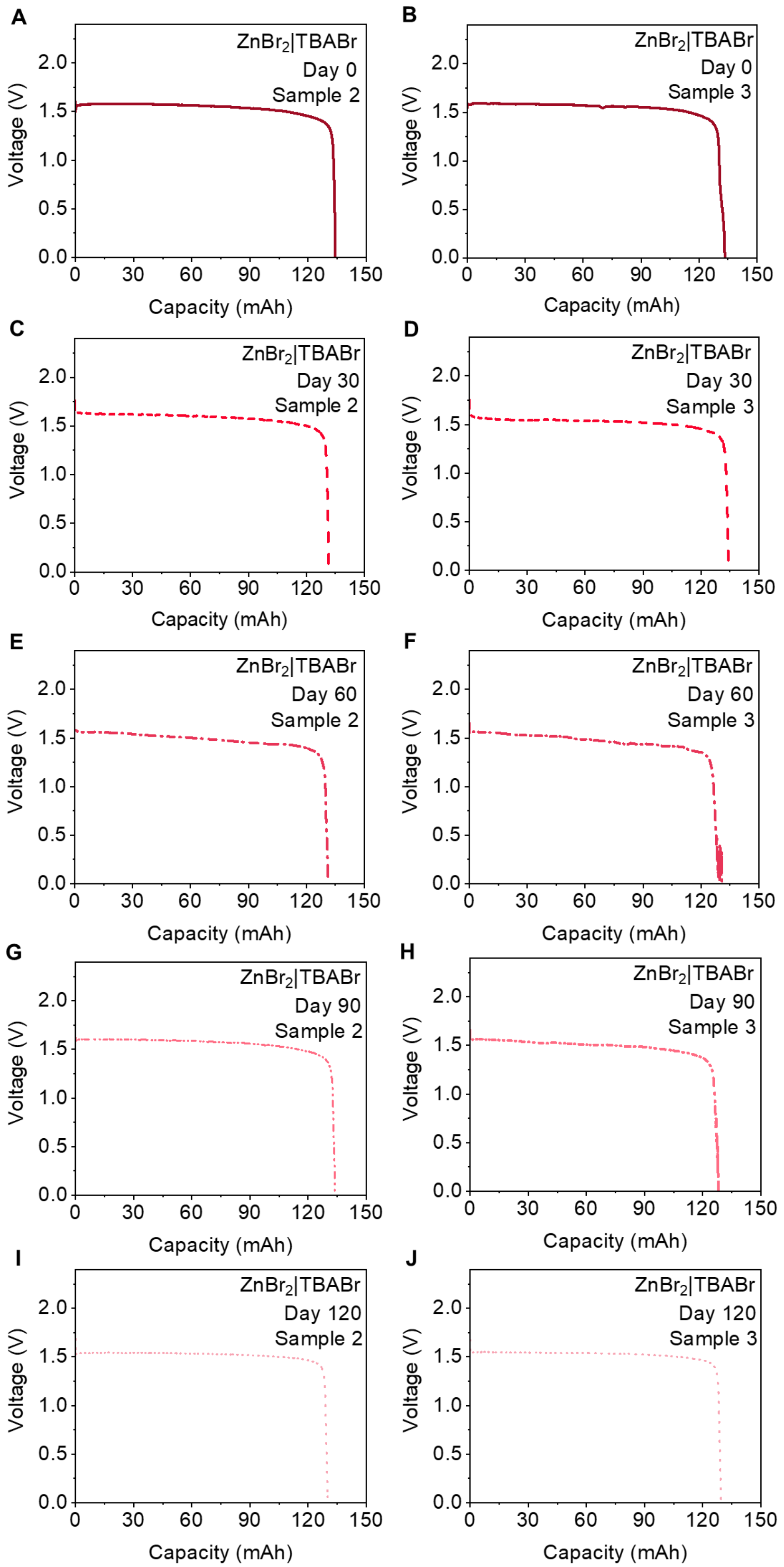

Figure S3. Repeated Days 0-120 capacity retention data of 0.5 M Z|T corresponding to Figure 2D.

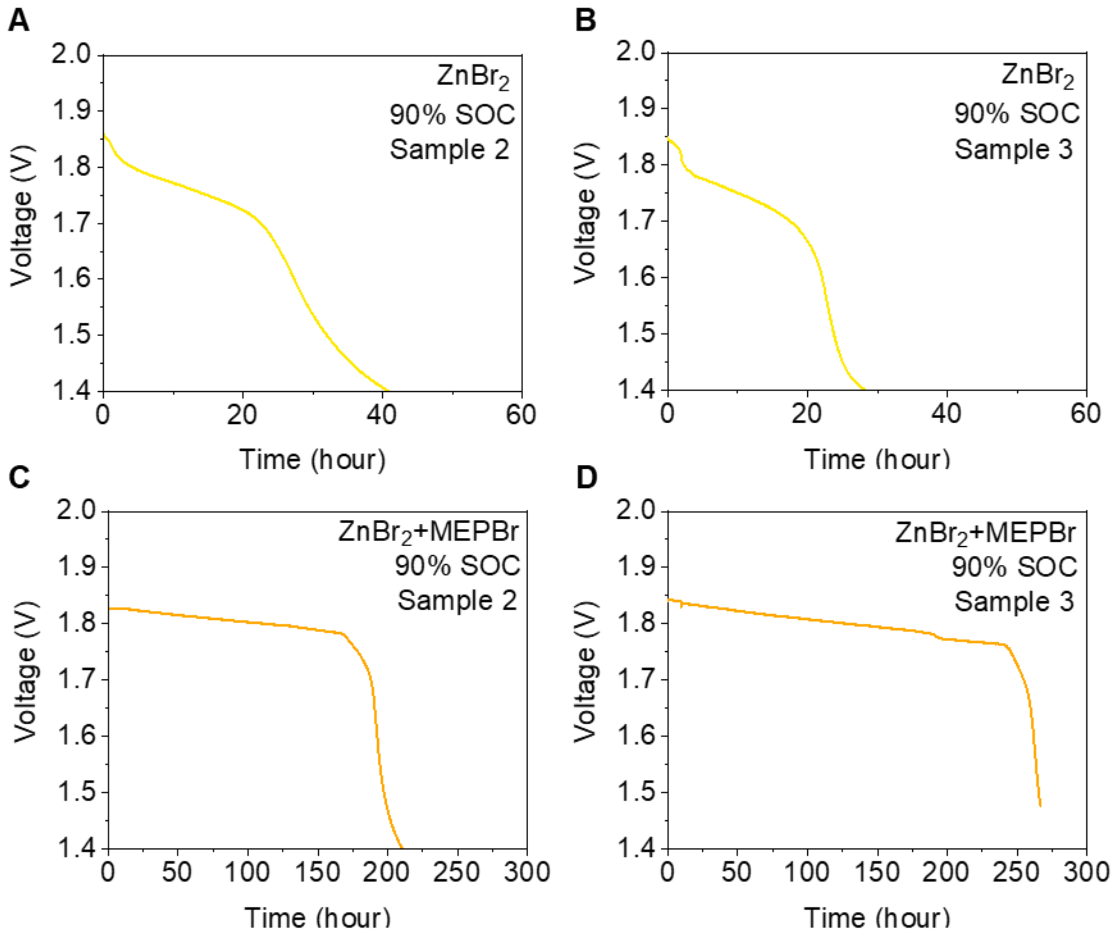

Figure S4. Repeated OCV retention of (A-B) $ZnBr_2$ and (C-D) $ZnBr_2$+MEPBr batteries at 90% SOC.

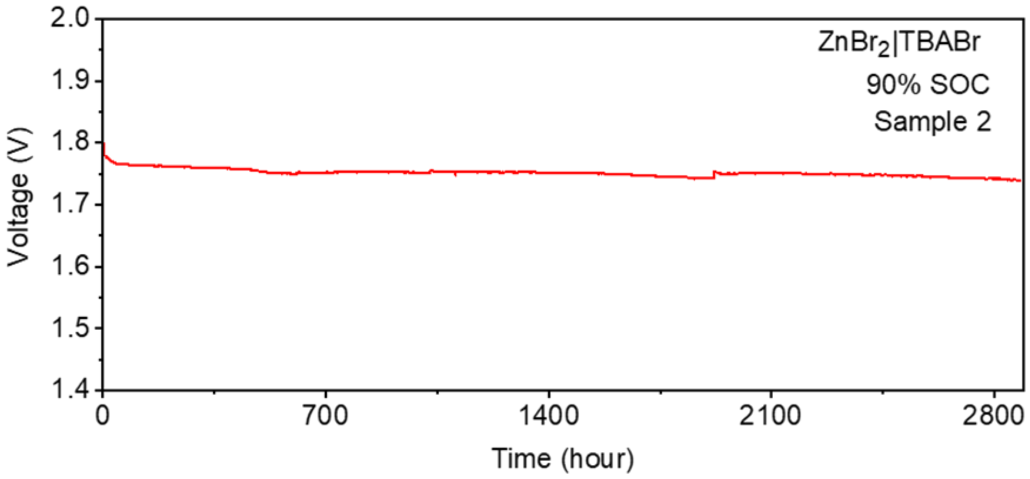

Figure S5. Repeated OCV retention of 0.5 M Z|T at 90% SOC corresponding to Figure 3A.

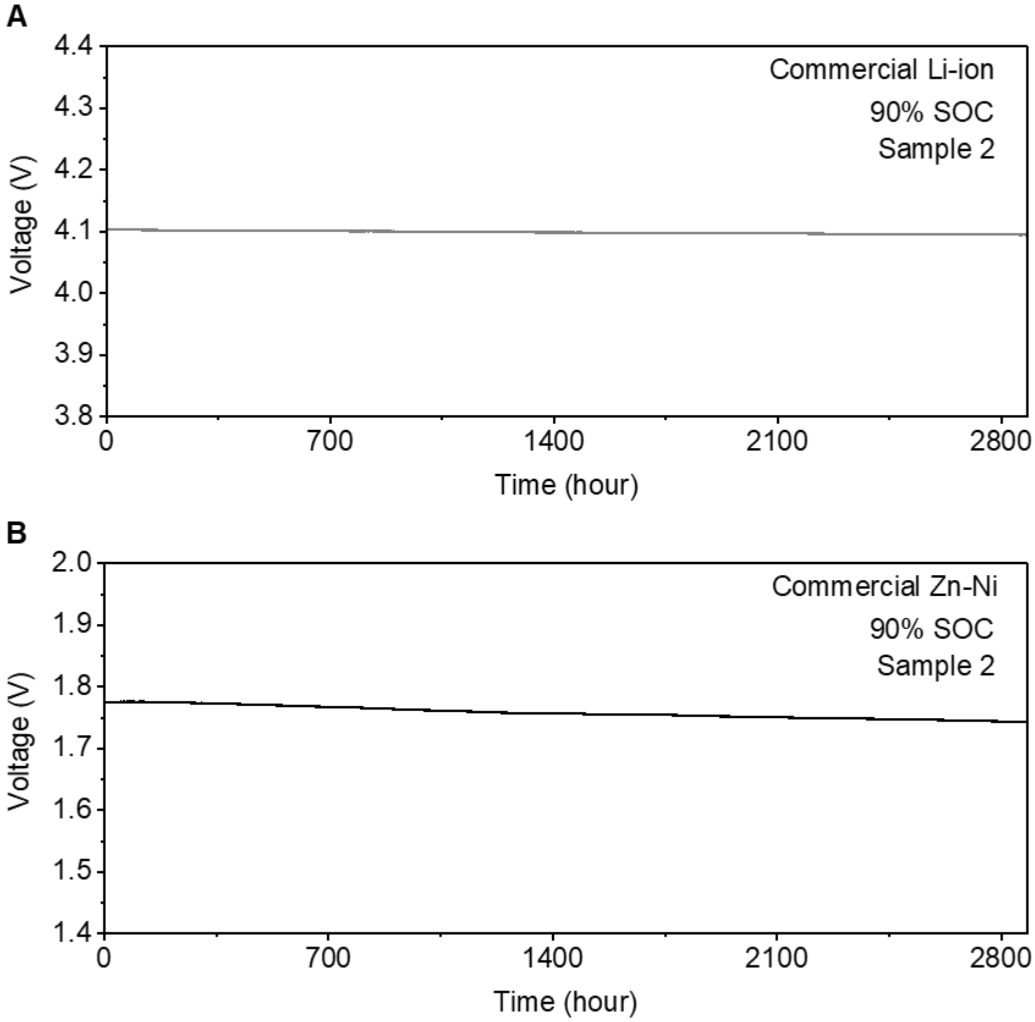

Figure S6. Repeated OCV retention of (A) commercial Li-ion and (B) Zn-Ni batteries at 90% SOC corresponding to Figure 3A.

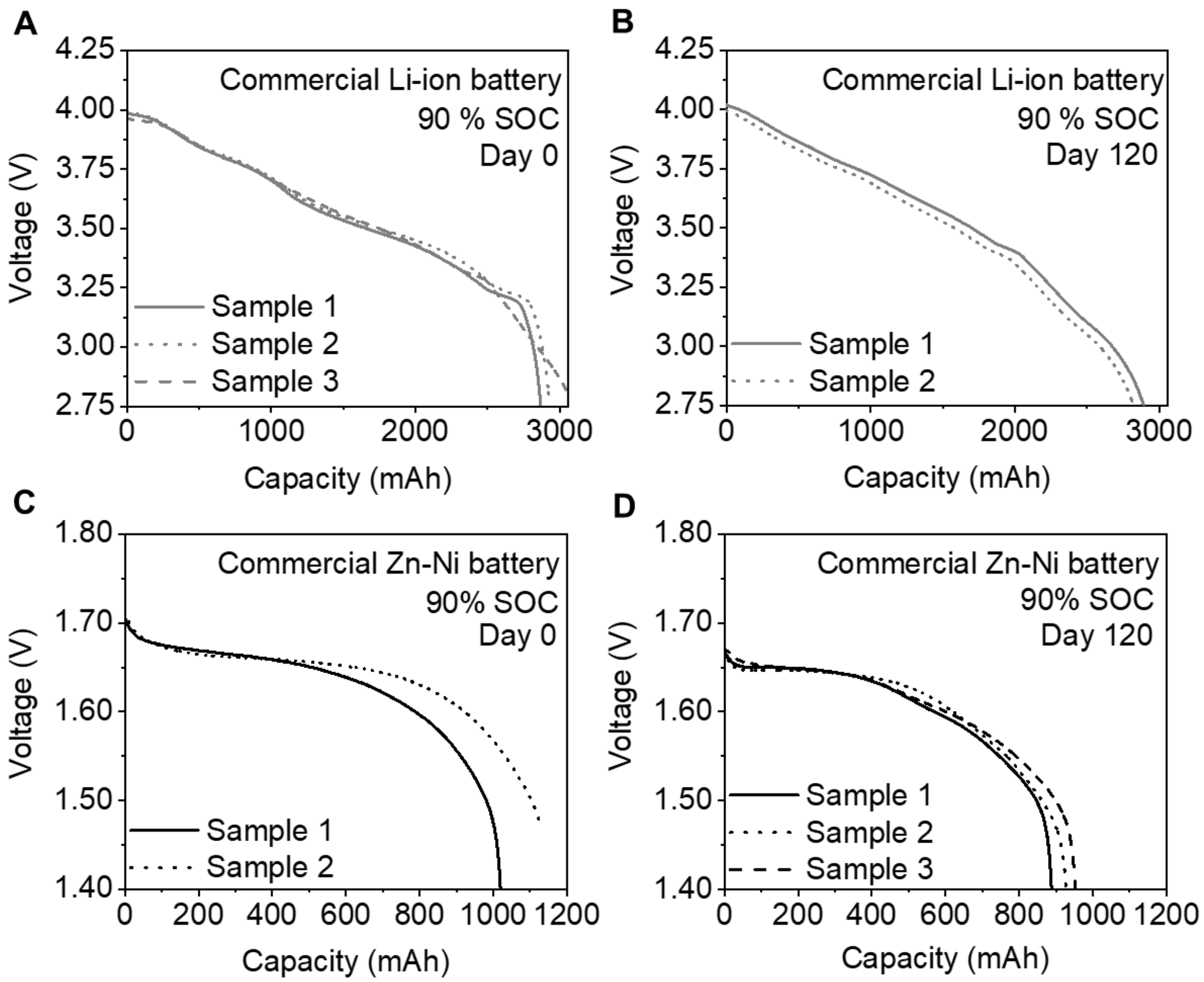

Figure S7. (A-B) Capacity retention of the commercial Li-ion batteries at 90% SOC on Days 0 and 120. (C-D) Capacity retention of the commercial Zn-Ni batteries at 90% SOC on Days 0 and 120.

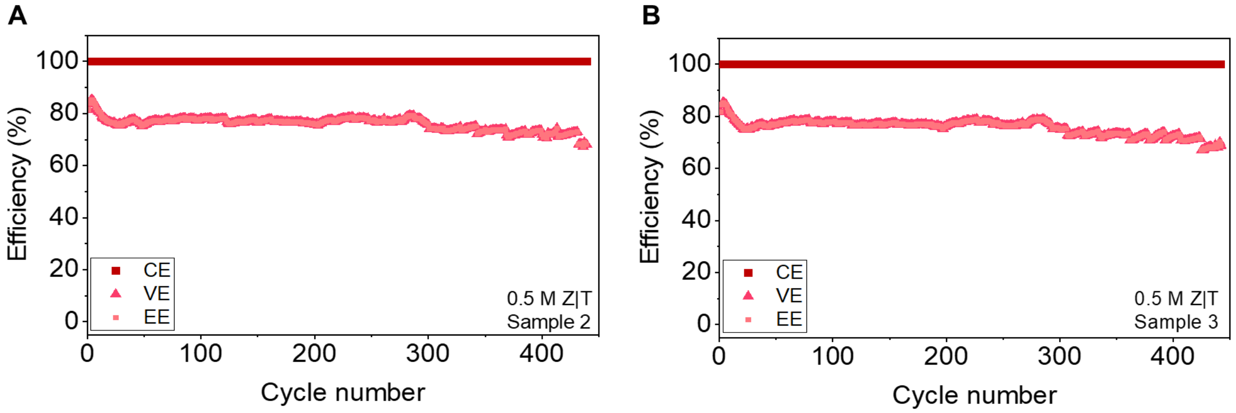

Figure S8. Repeated cycling performance of 0.5 M Z|T corresponding to Figure 4A.

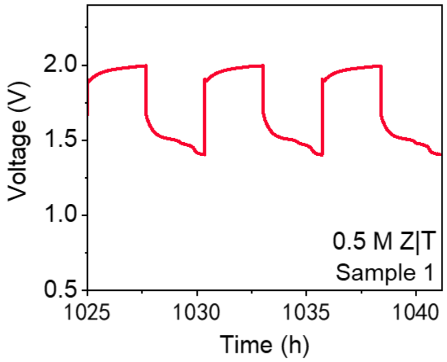

Figure S9. Voltage profile of the 0.5 M Z|T battery corresponding to Figure 4A.

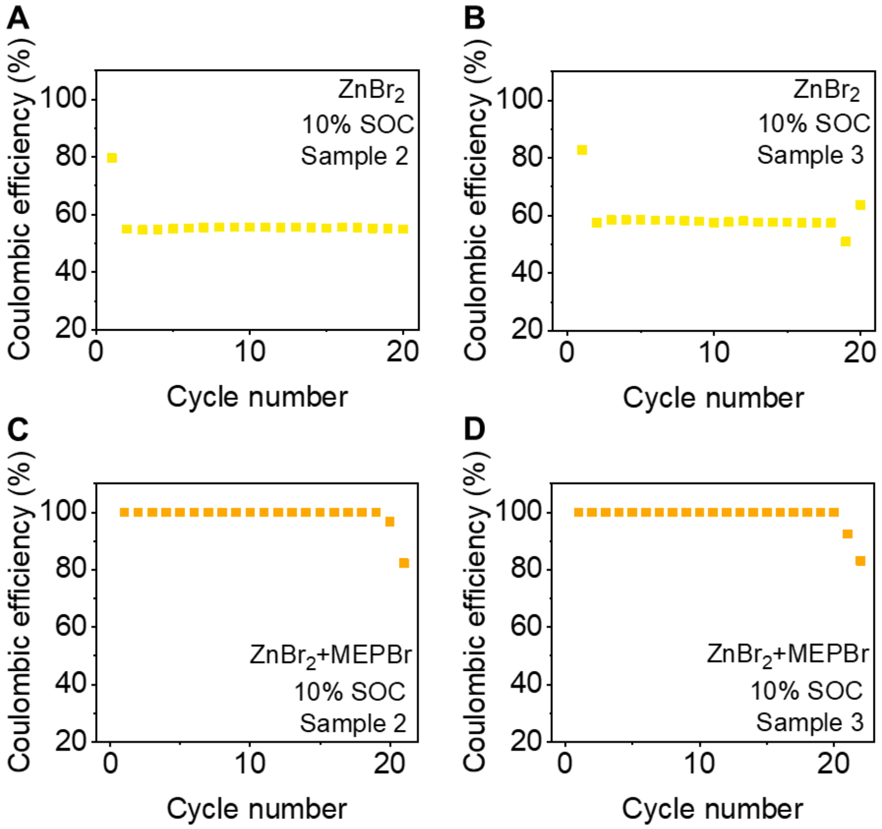

Figure S10. Coulombic efficiencies of (A-B) $ZnBr_2$ and (C-D) $ZnBr_2$+MEPBr batteries corresponding to Figure 4A.

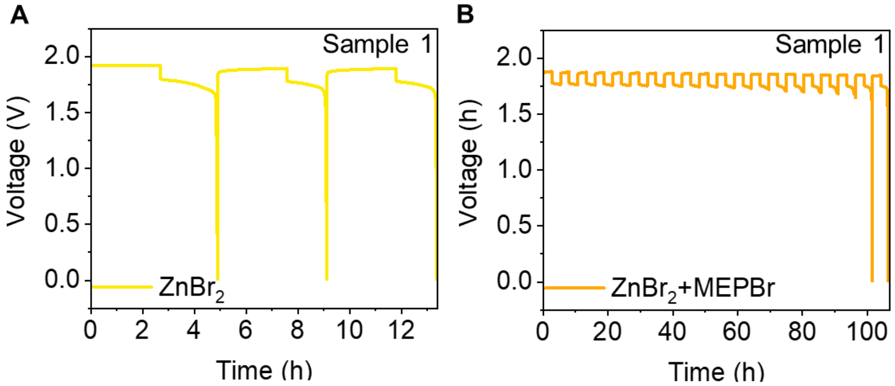

Figure S11. Voltage profiles of the (A) $ZnBr_2$ and (B) $ZnBr_2$+MEPBr batteries corresponding to Figure 4A.

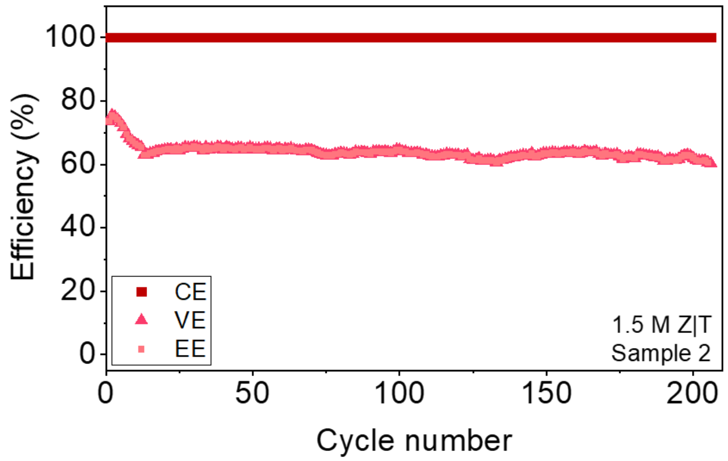

Figure S12. Repeated cycling performance of 1.5 M Z|T corresponding to Figure 4B.

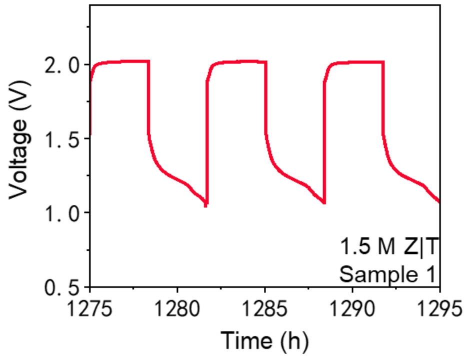

Figure S13. Voltage profile of the 1.5 M Z|T battery corresponding to Figure 4B.

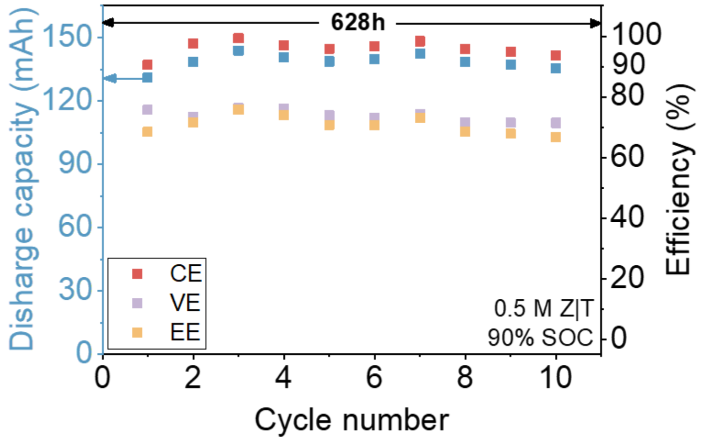

Figure S14. Repeated cycling performance of 0.5 M Z|T (90% total capacity for each cycle).

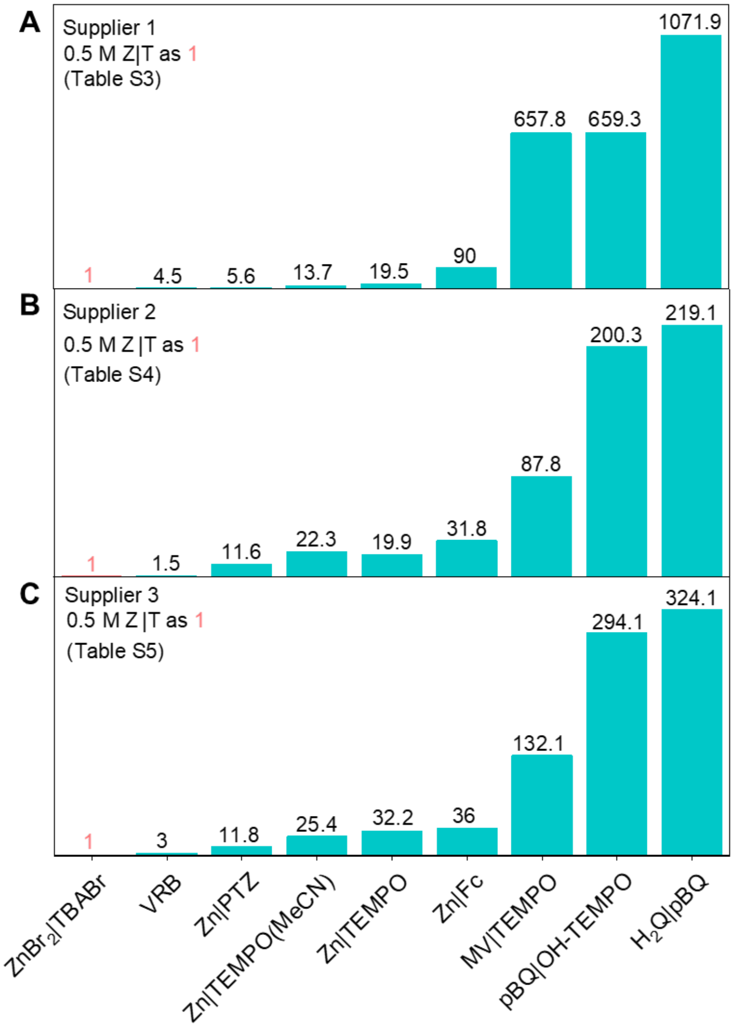

Figure S15. Cost comparison of Z|T to vanadium and other membraneless biphasic batteries from suppliers 1-3 corresponding to (A) Table S3 (B) Table S4 (C) Table S5.

Table S1. Details of previously reported liquid based batteries at various SOCs corresponding to Figure 3C.

Battery redox*	State of charge (%)	Time (h)	OCV drop (V)	Ref
**$ZnBr_2$|TBABr (Membraneless)**	**90**	**2880**	**1.777→1.735**	**This work**
Vanadium RFB (VRFB)	75	58	1.5→0.85	*[27]*
Polysulfide/Polyiodide RFB	50	900	Nearly no drop	*[28]*
VRFB	Charge to 1.6 V	24	1.6→0.8	*[29]*
VRFB	75	66	1.5→0.85	*[30]*
VRFB	/	290	1.6→1.3	*[31]*
VRFB	50	265	1.43→1.0	*[32]*
VRFB	50	160	1.3→0.8	*[33]*
VRFB	/	100	1.4→1.1	*[34]*
VRFB	100	13	1.35→1	*[35]*
VRFB	Charge to 1.6 V	184	1.6→1.3	*[36]*
VRFB	Charge to 1.65 V	85	1.65→1.4	*[37]*
VRFB	50	30	1.4→0.8	*[38]*
VRFB	50	40	1.4→1.25	*[39]*
VRFB	/	402	1.5→1.3	*[40]*
VRFB	/	140	1.5→1.3	*[41]*
VRFB	50	300	1.4→1.35	*[42]*
VRFB	75	120	1.5→1.3	*[43]*
Zn-Br RFB	/	13.4	1.77→1.69	*[44]*
VRFB	/	60	1.47→0.8	*[45]*
VRFB	50	260	1.45→0.8	*[46]*
Zn-Fe RFB	70	21	1.5→0.5	*[47]*
VRFB	/	30	1.5→0.8	*[48]*
VRFB	100	120	1.65→0.8	*[49]*
VRFB	/	239.9	1.5→0.8	*[50]*
VRFB	/	68	1.5→0.8	*[51]*
VRFB	100	384	1.66→1.5	*[52]*
VRFB	/	27	1.4→1.3	*[53]*
VRFB	Charge to 1.75 V	75	1.6→0.8	*[54]*
VRFB	75	68	1.5→0.85	*[55]*
VRFB	75	252	1.5→0.85	*[56]*
VRFB	75	73	1.5→0.8	*[57]*
VRFB	50	35	1.42→0.8	*[58]*
VRFB	/	202	1.5→0.8	*[59]*
VRFB	50	26.7	1.65→0.8	*[60]*
VRFB	/	39.78	1.46→0.8	*[61]*
VRFB	/	66	1.6→1.3	*[62]*
VRFB	50	300	1.42→0.8	*[63]*
VRFB	/	184	1.56→0.8	*[64]*
VRFB	Charge to 1.65 V	232	1.65→0.8	*[65]*
VRFB	75	231	1.3→0.8	*[66]*
VRFB	Charge to 1.65 V	33.16	1.5→0.8	*[67]*
VRFB	50	91	1.42→0.8	*[75]*
VRFB	Charge to 1.6 V	68	1.6→1.2	*[69]*
VRFB	/	330	1.5→1.3	*[70]*
Fe-Cr RFB	50	108	1→0.2	*[71]*
VRFB	70	52.5	1.2→ 0.8	*[72]*

VRFB	/	140	1.5→0.85	*[73]*
VRFB	Charge to 1.7 V	30	1.77→1.3	*[74)*
VRFB	50	216	1.5→0.8	*[68]*
VRFB	75	132	1.65→0.8	*[76]*
VRFB	75	60	1.65→0.85	*[77]*
VRFB	100	80	1.8→1.4	*[78]*
VRFB	50	165	1.65→0.8	*[79]*
VRFB	60	350	1.44→0.8	*[80]*
VRFB	Charge to 1.65 V	60	1.65→1.4	*[81]*
VRFB	50	41.67	1.4→0.8	*[82]*
VRFB	50	74	1.4→0.8	*[83]*
VRFB	75	35	1.56→0.75	*[84]*
VRFB	50	67	1.4→0.8	*[85]*
VRFB	50	350	1.5→0.85	*[86]*
VRFB	50	73	1.45→0.8	*[87]*
VRFB	75	60	1.5→0.8	*[92]*
VRFB	100	195	1.6→0.8	*[89]*
VRFB	50	98	1.4→0.8	*[90]*
VRFB	50	86.09	1.42→0.8	*[91]*
VRFB	75	76	1.44→0.8	*[88]*
Quinone-based RFB	75	120	0.56→0.38	*[93]*
VRFB	/	400	1.5→0.8	*[94]*
VRFB	50	231	1.42→0.8	*[95]*
VRFB	100	10.09	1.5→0.8	*[96]*
VRFB	/	576	1.6→0.8	*[97]*
VRFB	100	97	1.42→0.8	*[98]*
VRFB	Charge to 1.7 V	35	1.7→1.4	*[99]*
VRFB	50	136	1.44→0.8	*[100]*
VRFB	/	381	1.44→0.8	*[101]*
All-Heteropolyacid RFB	/	95	1.3→0.9	*[102]*
VRFB	100	52	1.6→1.25	*[103]*
VRFB	50	107	1.4→0.8	*[104]*
VRFB	50	160	1.4→0.8	*[105]*
VRFB	Charge to 1.65 V	418	1.65→0.8	*[106]*
VRFB	Charge to 1.58 V	124.5	1.58→0.8	*[107]*
VRFB	/	500	1.5→0.8	*[108]*
VRFB	/	595	1.5→0.8	*[109]*
VRFB	/	441	1.5→0.8	*[110]*
VRFB	50	180	1.4→0.8	*[111]*
VRFB	100	102	1.52→0.8	*[112]*
VRFB	/	84	1.5→0.8	*[113]*
VRFB	/	41.4	1.4→0.8	*[114]*
VRFB	75	44	1.4→0.8	*[115]*
VRFB	100	19	1.3→1.0	*[116]*
VRFB	75	87	1.5→0.8	*[117]*
VRFB	50	15.6	1.4→0.8	*[118]*
VRFB	100	21.21	1.6→0.8	*[119]*
VRFB	Charge to 1.6 V	300	1.6→1.3	*[120]*
VRFB	/	72	1.46→0.8	*[121]*
VRFB	Charge to 1.6 V	27.5	1.6→0.8	*[122]*

VRFB	100	107	1.6→0.8	*[123]*
VRFB	75	71	1.45→0.8	*[124]*
VRFB	50	109	1.35→0.8	*[125]*
VRFB	50	160	1.6→0.8	*[126]*
MV|TEMPO (Membraneless)	20	4.33	1.24→0.72	*[127]*
Zn|TEMPO (Membraneless)	100	100	1.6→1.52	*[26]*

*The batteries are with membrane unless marked with (Membraneless).

Table S2. Details of theoretical capacity, capacity achieved, and cycling performance of other biphasic membraneless batteries corresponding to Figure 4C.

Battery redox	Solvent (Active species)	Battery type	CE (%)	Capacity (Ah/L)	Cycling duration (hour)	Number of cycles	Ref
**$ZnBr_2$|TBABr**	**H_2O (0.5 M $ZnBr_2$) CH_2Cl_2 (0.5 M TBABr)**	**static**	**100**	**2.68**	**2670**	**500**	**This work**
**$ZnBr_2$|TBABr**	**H_2O (1.5 M $ZnBr_2$) CH_2Cl_2 (0.8 M TBABr)**	**static**	**100**	**8.04**	**1715**	**256**	**This work**
Zn|PTZ	H_2O (0.5 M $ZnSO_4$) CH_2Cl_2 (0.5 M C8-PTZ)	static	96	9.7→7.7	400	202	*[130]*
Zn|TEMPO	H_2O ($ZnSO_4$) TEGDME (TEMPO)	self-stratified	99	7.5	1000	500	*[132]*
H_2Q|pBQ	H_2O (0.1 M H_2Q) $PYR_{14}TFSI$ (0.1 M pBQ)	static	99	0.125	75	75	*[133]*
Zn|Fc	H_2O (1 M $ZnCl_2$) Butylacetate (0.1 M Fc)	static	78	0.024 → 0.026	12	20	*[131]*
DMFc	TFT/DCE (0.1 M DMFc) TFT/DCE (0.1 M DMFcTB)	flow	99	1.34	N/A	20	*[135]*
All-iron	H_2O (0.1 M $FeSO_4$) ethylacetate+$PYR_{14}TFSI$ (0.1 M $Fe(acac)_3$)	flow	80	1.34	N/A	25	*[129]*
pBQ| OH-TEMPO	H_2O (0.1 M OH-TEMPO) 75 wt% PC+ 25 wt% $PYR_{14}TFSI$ (0.1 M pBQ)	static	75	0.134	399	300	*[134]*
MV|TEMPO	25 wt% PEG1000+ 6 wt% Na_2SO_4+69 wt% H_2O (0.1 M MV, 0.1 M TEMPO)	static	82	0.134	/	550	*[127]*
Zn|TEMPO	H_2O (0.5 M $ZnSO_4$) MeCN (0.5 M TEMPO)	flow	99	7→12.3 →7	380	190	*[26]*

Table S3. Detailed prices and cost analysis of other biphasic membraneless and vanadium flow batteries from supplier 1[2].

**Battery redox**	**Price of each component**						**Cost ($/kWh)**	**Ref**
**$ZnBr_2$|TBABr (0.5 M)**	Component	$ZnBr_2$	TBABr	CH_2Cl_2			**1079**	**This work**
	Concentration (mol/L)	0.5	0.5	6 (mL)				
	Unit price ($/kg)	322.465	45.617	9.816 ($/L)				
**$ZnBr_2$|TBABr (1.5 M)**	Component	$ZnBr_2$	TBABr	CH_2Cl_2			**1004**	**This work**
	Concentration	1.5	0.8	9.375 (mL)				
	Unit price	322.465	45.617	9.816 ($/L)				
Zn|PTZ	Component	PTZ	$TBAPF_6$	CH_2Cl_2	$ZnSO_4$	KPF_6	6083	*[130]*
	Concentration	0.5	0.5	2 (mL)	0.5	0.3		
	Unit price	37.72	373.12	9.816($/L)	14.79	44.74		
Zn|TEMPO	Component	TEMPO	TEGDME	LiTFSI	$ZnSO_4$	$MgSO_4$	21025	*[132]*
	Concentration	0.18 (g)	2.0 (g)	0.74 (g)	0.72 (g)	1.08 (g)		
	Unit price	308.31	61.74	891.89	14.79	61.35		
H_2Q|pBQ	Component	H_2Q	HCl (37%)	pBQ	$PYR_{14}TFSI$		1156615	*[133]*
	Concentration	0.1	0.1	0.1	10 (mL)			
	Unit price	84.94	24.85 ($/L)	132.13	6197.62			
Zn|Fc	Component	$ZnCl_2$	Fc	Butylacetate	Aliquat336		97124	*[131]*
	Concentration	1	0.1	3.5 (mL)	1.5 (mL)			
	Unit price	202.92	72.36	31.3 ($/L)	95.39 ($/L)			
DMFc	Volume of each solvent not provided						N/A	*[135]*
All-iron	Actual operating volume not clarified						N/A	*[129]*
pBQ|OH-TEMPO	Component	PC	$PYR_{14}TFSI$	OH-TEMPO	NaCl	pBQ	711412	*[134]*
	Concentration	8.4 (g)	2.8 (g)	0.1	0.5	0.1		
	Unit price	45.62	6197.62	308.31	9.75	123.13		
MV|TEMPO	Component	TEMPO	MV	PEG1000	Na_2SO_4		709736	*[127]*
	Concentration	0.1	0.1	2.53 (g)	0.61 (g)			
	Unit price	308.31	9026.67	16.36	9.438			
Zn|TEMPO	Component	$ZnSO_4$	$MgSO_4$	NH_4+TBA PF_6	MeCN	TEMPO	14784	*[26]*
	Concentration	0.5	1.8	0.3+0.5	2 (mL)	0.5		
	Unit price	14.79	61.35	313+373.1	29.57 ($/L)	308.31		
VRFB	Component	$VOSO_4$	H_2SO_4 (8N)				3529	3
	Concentration	3	3					
	Unit price	725.153	79.3 ($/L)					

Table S4. Detailed prices and cost analysis of other biphasic membraneless and vanadium flow batteries from supplier 2[4].

Battery redox	Price of each component						Cost ($/kWh)	Ref
**$ZnBr_2$|TBABr (0.5 M)**	Component	$ZnBr_2$	TBABr	CH_2Cl_2			**5602**	**This work**
	Concentration (mol/L)	0.5	0.5	6 (mL)				
	Unit price ($/kg)	476	918	76.2 ($/L)				
**$ZnBr_2$|TBABr (1.5 M)**	Component	$ZnBr_2$	TBABr	CH_2Cl_2			**5026**	**This work**
	Concentration	1.5	0.8	9.375 (mL)				
	Unit price	476	918	76.2 ($/L)				
Zn|PTZ	Component	PTZ	$TBAPF_6$	CH_2Cl_2	$ZnSO_4$	KPF_6	65056	*[130]*
	Concentration	0.5	0.5	2 (mL)	0.5	0.3		
	Unit price	114.6	2050	76.2 ($/L)	545	436		
Zn|TEMPO	Component	TEMPO	TEGDME	LiTFSI	$ZnSO_4$	$MgSO_4$	111486	*[132]*
	Concentration	0.18 (g)	2.0 (g)	0.74 (g)	0.72 (g)	1.08 (g)		
	Unit price	7120	222.8	3980	448	242		
H_2Q|pBQ	Component	H_2Q	HCl (37%)	pBQ	$PYR_{14}TFSI$		1227196	*[133]*
	Concentration	0.1	0.1	0.1	10 (mL)			
	Unit price	306	119.8 ($/L)	418	9200			
Zn|Fc	Component	$ZnCl_2$	Fc	Butylacetate	Aliquat336		178375	*[131]*
	Concentration	1	0.1	3.5 (mL)	1.5 (mL)			
	Unit price	308	408	180 ($/L)	166.1 ($/L)			
DMFc	Volume of each solvent not provided						N/A	*[135]*
All-iron	Actual operating volume not clarified						N/A	*[129]*
pBQ|OH-TEMPO	Component	PC	$PYR_{14}TFSI$	OH-TEMPO	NaCl	pBQ	1122143	*[134]*
	Concentration	8.4 (g)	2.8 (g)	0.1	0.5	0.1		
	Unit price	149.8	9200	7120	95.2	418		
MV|TEMPO	Component	TEMPO	MV	PEG1000	Na_2SO_4		491788	*[127]*
	Concentration	0.1	0.1	2.53 (g)	0.61 (g)			
	Unit price	7120	56300	54.3	131			
Zn|TEMPO	Component	$ZnSO_4$	$MgSO_4$	NH_4+TBA PF_6	MeCN	TEMPO	124671	*[26]*
	Concentration	0.5	1.8	0.3+0.5	2 (mL)	0.5		
	Unit price	448	242	2730+2050	135 ($/L)	7120		
VRFB	Component	$VOSO_4$	H_2SO_4 (10N)				6384	3
	Concentration	3	3					
	Unit price	1312	34 ($/L)					

Table S5. Detailed prices and cost analysis of other biphasic membraneless and vanadium flow batteries from supplier 3[5].

**Battery redox**	**Price of each component**						**Cost ($/kWh)**	**Ref**
**$ZnBr_2$|TBABr (0.5 M)**	Component	$ZnBr_2$	TBABr	CH_2Cl_2			**3733**	**This work**
	Concentration (mol/L)	0.5	0.5	6 (mL)				
	Unit price ($/kg)	412.7	549.2	48.3 ($/L)				
**$ZnBr_2$|TBABr (1.5 M)**	Component	$ZnBr_2$	TBABr	CH_2Cl_2			**3368**	**This work**
	Concentration	1.5	0.8	9.375 (mL)				
	Unit price	412.7	549.2	48.3 ($/L)				
Zn|PTZ	Component	PTZ	$TBAPF_6$	CH_2Cl_2	$ZnSO_4$	KPF_6	43870	*[130]*
	Concentration	0.5	0.5	2 (mL)	0.5	0.3		
	Unit price	113.44	1680	48.3 ($/L)	74.56	652.1		
Zn|TEMPO	Component	TEMPO	TEGDME	LiTFSI	$ZnSO_4$	$MgSO_4$	120039	*[132]*
	Concentration	0.18 (g)	2.0 (g)	0.74 (g)	0.72 (g)	1.08 (g)		
	Unit price	7938	170.96	4410	74.56	130.62		
H_2Q|pBQ	Component	H_2Q	HCl (37%)	pBQ	$PYR_{14}TFSI$		1209795	*[133]*
	Concentration	0.1	0.1	0.1	10 (mL)			
	Unit price	98.28	239.4 ($/L)	298.2	9072			
Zn|Fc	Component	$ZnCl_2$	Fc	Butylacetate	Aliquat336		134553	*[131]*
	Concentration	1	0.1	3.5 (mL)	1.5 (mL)			
	Unit price	258.3	303.24	67.84 ($/L)	166.1 ($/L)			
DMFc	Volume of each solvent not provided						N/A	*[135]*
All-iron	Actual operating volume not clarified						N/A	*[129]*
pBQ|OH-TEMPO	Component	PC	$PYR_{14}TFSI$	OH-TEMPO	NaCl	pBQ	1097866	*[134]*
	Concentration	8.4 (g)	2.8 (g)	0.1	0.5	0.1		
	Unit price	108.8	9072	7938	32.24	298.2		
MV|TEMPO	Component	TEMPO	MV	PEG1000	Na_2SO_4		493080	*[127]*
	Concentration	0.1	0.1	2.53 (g)	0.61 (g)			
	Unit price	7938	56300	63.21	45.15			
Zn|TEMPO	Component	$ZnSO_4$	$MgSO_4$	NH_4+TBA PF_6	MeCN	TEMPO	94905	*[26]*
	Concentration	0.5	1.8	0.3+0.5	2 (mL)	0.5		
	Unit price	74.56	130.62	1785+1680	75.7 ($/L)	7938		
VRFB	Component	$VOSO_4$	H_2SO_4 (6N)				8272	3
	Concentration	3	2					
	Unit price	1700	51.03 ($/L)					

Table S6. Detailed prices and cost analysis of $ZnBr_2$|TBABr batteries in industry grade.

Battery redox	Price of each component				Cost ($/kWh)	Ref
**$ZnBr_2$|TBABr (0.5 M)**	Component	$ZnBr_2$	TBABr	CH_2Cl_2	**21.6**	**This work**
	Concentration (mol/L)	0.5	0.5	6 (mL)		
	Unit price ($/kg)	1.89	3.15	0.199 ($/L)		
**$ZnBr_2$|TBABr (1.5 M)**	Component	$ZnBr_2$	TBABr	CH_2Cl_2	**18.9**	**This work**
	Concentration	1.5	0.8	9.375 (mL)		
	Unit price	1.89	3.15	0.199 ($/L)		

*The unit price of materials was obtained from https://www.1688.com/ (Feb 2023)

Supplementary movies

Movie S1. KI paper test on DI water, bromine water (1.0 & 0.2 mM) and the aqueous phase of 0.5 M Z|T (Days 0 & 120) at 90% SOC, corresponding to Figure 2B.

SI References

1. X. Shi *et al.*, Polymer Electrolyte Membranes for Vanadium Redox Flow Batteries: Fundamentals and Applications. *Progress in Energy and Combustion Science* **85**, 100926 (2021).
2. Aladdin, Data of chemicals and reagents prices from July 2022. https://www.aladdin-e.com/.
3. C. Choi *et al.*, A review of vanadium electrolytes for vanadium redox flow batteries. *Renewable and Sustainable Energy Reviews* **69**, 263-274 (2017).
4. Sigma-Aldrich, Data of chemicals and reagents prices from July 2022. https://www.sigmaaldrich.com/US/en.
5. Alfa Aesar, Data of chemicals and reagents prices from July 2022. https://www.alfa.com/zh-cn/.
